# Synthetic, Biochemical, Antifertility and Antiinflammatory Aspects of Manganese and Iron Complexes

**DOI:** 10.1155/MBD.2002.97

**Published:** 2002

**Authors:** Ashu Chaudhary, Anita Phor, Sanjay Sharma, Anita Gajraj, R. V. Singh

**Affiliations:** 1 Department of Chemistry University of Rajasthan Jaipur 302004 India; 2 Hindu College Sonipat 131001 India; 3 Institute of Engineering and Technology, M.I.A. Alwar India; 4 Department of Zoology University of Rajasthan Jaipur 302 004 India

## Abstract

Manganese(II) and iron(II) macrocyclic complexes of polyamide groups have been synthesized by the template codensation of diamines (2,6 diaminopyridine, 1,2 phenylenediamine and 1,3 phenylenediame) and triamine (diethylenetriamine) with phthalic acid in 1:2:2 molar ratios. On the basis of elemental analysis, IR, electronic, magnetic moment, Mössbauer, mass and X-ray spectral studies, octahedral structure has been assigned to $\left[\text{M}\left({\text{N}}_{4}{\text{mac}}^{\text{n}}\right){\text{Cl}}_{2}\right]$ (M = Mn(II) and Fe(II), n = 1 to 4) complexes. The complexes have been screened *in vitro* against a number of fungi and bacteria to assess their growth inhibiting potential. An attempt has been made to correlate the structural aspects of the compounds with their antiinflammatory and antifertility activities.


### Synthetic, Biochemical, Antifertility and Antiinflammatory Aspects of Manganese and Iron Complexes

Ashu Chaudhary[1], Anita Phor[2], Sanjay Sharma[3], Anita Gajraj[4] and R.V. Singh[1]*

[1]*Department of Chemistry, University of Rajasthan, Jaipur – 302004, India.*
[2]*Hindu College, Sonipat - 131001*
[3]*Institute of Engineering and Technology, M.I.A. Alwar, India*
[4]*Department of Zoology, University of Rajasthan, Jaipur – 302 004, India*
*E-mail : kudiwal@datainfosys.net; Fax : +91(141)708621*

### ABSTRACT

Manganese(II) and iron(II) macrocyclic complexes of polyamide groups have been synthesized by the template condensation of diamines (2,6 diaminopyridine, 1,2 phenylenediamine and 1,3 phenylenediame) and triamine (diethylenetriamine) with phthalic acid in 1:2:2 molar ratios. On the basis of elemental analysis, IR, electronic, magnetic moment, Mössbauer, mass and X-ray spectral studies, octahedral structure has been assigned to $[M(N_4mac^n)Cl_2]$ (M = Mn(II) and Fe(II), n = 1 to 4) complexes. The complexes have been screened *in vitro* against a number of fungi and bacteria to assess their growth inhibiting potential. An attempt has been made to correlate the structural aspects of the compounds with their antiinflammatory and antifertility activities.

### INTRODUCTION

The field of macrocyclic compounds is developing very fast due to their variety of applications /1/ and importance in the area of coordination chemistry /2/. The recognition of a metal ion by a macrocyclic ligand and modification of the properties of the resulting complexes is controlled to a large extent by a match between the size of the ligand hole and that of the metal ion /3/. The very high thermodynamic stability and extreme kinetic inertness of transition metal complexes of polyazamacrocyclic ligands are significant since they enhance a number of industrial applications /4/. A review on macrocycles has revealed the importance of macrocyclic complexes in biological processes such as photosynthesis and dioxygen transport /5/, their catalytic properties /6/, their potential applications as metal extractants and as radio-therapeutic /7/ and medical imaging agents. Macrocyclic polyamines have attracted increasing attention because of their unique property, namely to form very stable chelates with various heavy metal ions /8/. Studies on the polyazamacrocycles, particularly the tetraaza groups have also been published frequently, particularly in view of their potential for binding more than one metal ion /9/.

Macrocyclic complexes of manganese and iron are used as antimicrobial, antifertility, antiinflammatory and analgesic agents. Manganese, together with copper and iron, are essential metallic elements and exhibit sufficient biological activity, when associated with certain metal protein complexes participating in the storage of ions /10/, to create enormous interest in the study of systems containing these metals /11/. A detailed study of the interaction of Mn(II) and Fe(II) with diclofenac sodium (non-steriodal antiinflammatory drug was undertaken by Singh *et al.* /12/. Chondhekar *et al.* /13/ have reported the fungitoxic studies of Mn(II) with heterocyclic Schiff base ligand, due to its wide applications in the food industry and agrochemical activity. Recently, Devereux /14/ and coworkers have shown that a range of carboxylate complexes incorportating manganese and cobalt metal centres inhibit the growth of Candida albicans /15/. Fe(II) complexes with 1,10-phenanthroline are known to have a wide spectrum of antimicrobial actions and to produce negligible toxicity to skin subcutaneous tissues and mucocus membranes /15/.

Mn(II) complexes of 3,4,7,8 tetramethyl-1, 10-phenthroline were used topically to treat patients suffering from a variety of skin conditions, many of whom had chronic dermatological infections due to dermatophytes (e.g. *malassezia furfur*, *trichophyton rubrum*) or candida species /16/. The complexes produced a significant decrease in microbial infection in approximately 50% of cases, with infection due to gram positive bacteria generally responding much more rapidly and readily to treatment than infection due to gram negative bacteria. Manganese chloride causes loss of testicular germ cells in rats and rabbits /16/ and decreased libido and impotency were noted in men occupationally exposed to manganese, but the data are inconclusive. The aim of the antifertility activity was to assess the effect on fertility and to contribute to a better understanding of the reproductive function of male albino rats. Hence, it was thought of considerable interest to synthesize macrocyclic complexes of manganese and iron with a view to evaluate their antimicrobial, antiinflammatory and antifertility activities.

### EXPERIMENTAL

All the chemicals used in the synthesis of the complexes were of AR grade. $MnCl_2.4H_2O$ and $FeCl_2.4H_2O$ (B.D.H.), phthalic acid (Fluka) and amines (E. Merck) were used as received.

### Synthesis of the Complexes $[Mn(N_4mac^1)Cl_2]$

The weighed amount of $MnCl_2.4H_2O$ (0.99 g/5.0 mmol) was dissolved in a minimum amount of methanol at 0°C and put in a magnetically stirred 100 mL round botton flask. The stirred solution of $MnCl_2.4H_2O$ was reacted with diethylenetriamine (1.04 g/10.0 mmol) dissolved in methanol. This was followed by the addition of a methanolic solution of phthalic acid (1.66 g/10 mmol). The reaction was carried out in 1:2:2 molar ratios. The resultant solid products were filtered, washed several times with methanol and dried. These compounds were recrystallized in benzene and dried again *in vacuo*.

Some other compounds $[M(N_4mac^n)Cl_2]$(M = Mn(II) and Fe(II), n = 2-4) were synthesized from various amines (2,6 diaminopyridine, 1,2-phenylene diamine and 1,3-phenylenediamine and $MnCl_2.4H_2O$ or $FeCl_2.4H_2O$ by keeping phthalic acid as constant reactant.

### Analytical Methods and Physical Measurements

The molecular weights were determined by the Rast camphor method. Conductivity measurements in dry dimethylformamide were performed with a conductivity bridge type 305. Infrared spectra were recorded on a Nicolet Magna FT.IR 550 spectrophotometer in KBr pellets. Electronic spectra in dimethylsulphoxide were recorded on a UV-160 A, Shimadzu spectrophotometer in the range 200-600 nm using methanol as a solvent. X-ray powder diffraction spectra of the compounds were obtained on the Philips model P.W. 1840 automatic diffractometer using $Fe(K_\alpha)$ target with Mg filter. The wavelength used was 1.9373 A° and the reflections from 5-65° were recorded. The mass spectra of the compounds were recorded on a JEOL FX 102/DA-6000 mass spectrometer/data system using Argon/Xenon (6 KV, 10 mA) as the FAB gas. m-Nitrobenzyl alcohol was used as the matrix. Manganese and iron were estimated gravimetrically. Carbon and hydrogen analyses were performed at Central Drug Research Institute, Lucknow.

### RESULTS AND DISCUSSION

The macrocyclic complexes have been prepared by the condensation reactions of phthalic acid and amines (diethylenetriamine, 2,6-diaminopyridine, 1,2-phenylenediamine and 1,3-phenylenediamine) and metal salt.

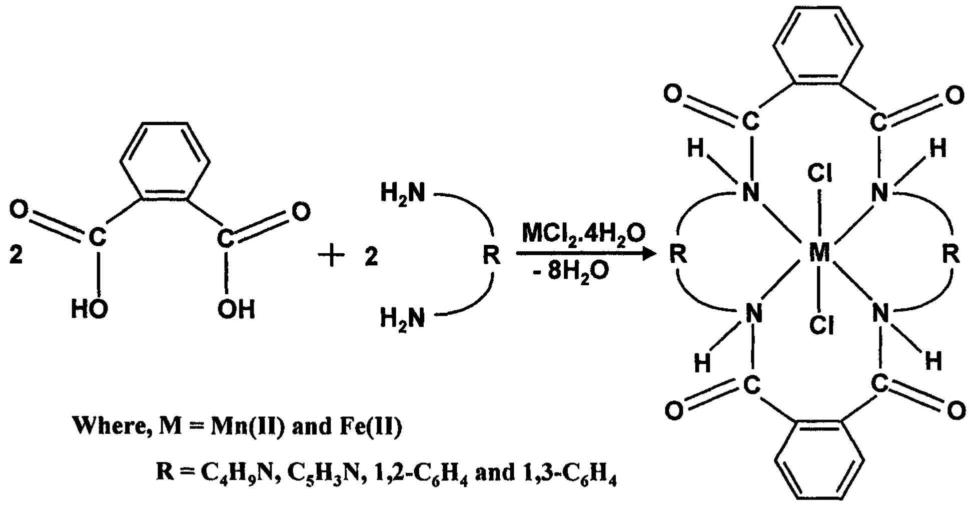

**Where, M = Mn(II) and Fe(II)**

**R = $C_4H_9N$, $C_5H_3N$, 1,2-$C_6H_4$ and 1,3-$C_6H_4$**

The resulting complexes are coloured solids, having sharp melting points. These are soluble in common organic solvents. The molar conductances of $10^{-3}$ M solutions of the compounds in anhydrous dimethylformamide lie in the range 5-29 $ohm^{-1}$ $cm^{2}$ $mol^{-1}$, which shows that the complexes are non-electrolytes and thus indicates that the anions are coordinated to the metal in these complexes.

The physical properties and analytical data of the complexes are listed in Table 1.

**Table 1**

Physical Properties and Analytical Data of Macrocyclic Complexes.

| Compound | Empirical formula and Colour | M.P. (°C) | Analysis % Found (Calcd.) C | H | N | Cl | M | Mol. Wt. Found (Calcd.) |
|---|---|---|---|---|---|---|---|---|
| $[Mn(N_4mac^1)Cl_2]$ | $C_{24}H_{30}O_4N_6Cl_2Mn$ | 119 | 48.28 | 4.96 | 12.89 | 11.39 | 8.18 | 568 |
| | Cream | | (48.69) | (5.11) | (14.19) | (11.98) | (9.2) | (592) |
| $[Mn(N_4mac^2)Cl_2]$ | $C_{26}H_{18}O_4N_6Cl_2Mn$ | 250 | 51.23 | 2.89 | 13.13 | 11.10 | 8.59 | 585 |
| | Dark brown | | (51.70) | (3.00) | (13.91) | (11.74) | (9.09) | (604) |
| $[Mn(N_4mac^3)Cl_2]$ | $C_{28}H_{20}O_4N_4Cl_2Mn$ | 179 | 55.46 | 3.22 | 8.53 | 11.13 | 8.59 | 593 |
| | Peach | | (55.89) | (3.35) | (9.31) | (11.78) | (9.12) | (602) |
| $[Mn(N_4mac^4)Cl_2]$ | $C_{28}H_{20}O_4N_4Cl_2Mn$ | 182 | 55.49 | 3.21 | 8.52 | 11.11 | 8.62 | 589 |
| | Brown | | (55.89) | (3.35) | (9.31) | (11.78) | (19.12) | (602) |
| $[Fe(N_4mac^1)Cl_2]$ | $C_{24}H_{30}O_4N_6Cl_2Fe$ | 198 | 48.22 | 4.99 | 12.57 | 11.25 | 8.70 | 562 |
| | Bright brown | | (48.61) | (5.10) | (14.17) | (11.95) | (9.40) | (593) |
| $[Fe(N_4mac^2)Cl_2]$ | $C_{26}H_{18}O_4N_6Cl_2Fe$ | 193 | 51.18 | 2.88 | 12.69 | 11.04 | 8.64 | 577 |
| | Green | | (51.62) | (3.00) | (13.89) | (11.72) | (9.23) | (605) |
| $[Fe(N_4mac^3)Cl_2]$ | $C_{28}H_{20}O_4N_4Cl_2Fe$ | 231 | 55.36 | 3.17 | 9.13 | 11.14 | 8.72 | 580 |
| | Peach | | (55.77) | (3.34) | (9.29) | (11.75) | (9.26) | (603) |
| $[Fe(N_4mac^4)Cl_2]$ | $C_{28}H_{20}O_4N_4Cl_2Fe$ | 167 | 55.36 | 3.15 | 8.30 | 11.11 | 8.79 | 578 |
| | Brown | | (55.77) | (3.34) | (9.29) | (11.75) | (9.26) | (603) |

### Spectral Studies

### *IR Spectra*

The characteristic infrared frequencies of the complexes are summarized in Table 2. The IR spectra of the metal complexes do not show the bands due to the $-OH$ group of phthalic acid and the $-NH_2$ group of amines, which indicates the condensation of these groups. The appearance of four amide bands in the regions 1632-1715, 1447-1587, 1240-1304 and 641-691 $cm^{-1}$ in plane deformation vibrations suggests the proposed cyclization /17/. A single sharp absorption, pointed in the region 3285-3340 $cm^{-1}$, is attributed to $\nu$(NH) of the amide group /18/. A new medium intensity band in the region 410-459 $cm^{-1}$ due to $\nu$(M-N) vibrations /19/ further confirms the involvement of nitrogen in coordination. All the complexes show a band in the region 285-370 $cm^{-1}$, assignable to $\nu$(M-Cl) modes /20/. The literature survey reveals that macrocyclic complexes of transition metals with similar ligands show coordination between the amide nitrogen and the central metal atom /21-24/.

**Table 2**

IR Spectral Data (in $cm^{-1}$) of the Macrocyclic Complexes

| Compound | ν(N-H) | Amide | | | | ν(C-N) | ν(M-N) | ν(M-Cl) |
|---|---|---|---|---|---|---|---|---|
| | | I | II | III | IV | | | |
| $[Mn(N_4mac^1)Cl_2]$ | 3285 | 1649 | 1447 | 1247 | 641 | - | 415 | 285 |
| $[Mn(N_4mac^2)Cl_2]$ | 3291 | 1632 | 1456 | 1264 | 685 | 852 | 433 | 344 |
| $[Mn(N_4mac^3)Cl_2]$ | 3332 | 1656 | 1577 | 1244 | 655 | - | 41 | 335 |
| $[Mn(N_4mac^4)Cl_2]$ | 3314 | 169 | 1546 | 1293 | 663 | - | 429 | 369 |
| $[Fe(N_4mac^1)Cl_2]$ | 3327 | 175 | 1587 | 1278 | 691 | - | 451 | 333 |
| $[Fe(N_4mac^2)Cl_2]$ | 3340 | 1686 | 1498 | 1284 | 680 | 849 | 448 | 370 |
| $[Fe(N_4mac^3)Cl_2]$ | 3337 | 1715 | 1539 | 1257 | 672 | - | 436 | 292 |
| $[Fe(N_4mac^4)Cl_2]$ | 3289 | 1702 | 1472 | 1304 | 649 | - | 459 | 298 |

***Electronic Spectra***

The electronic spectra of the Mn(II) complexes display weak absorption bands in the regions 16815-16859, 23503-23528 and 26338-26507 $cm^{-1}$ assignable to the transitions $^6A_{1g} \rightarrow {}^4T_{1g}$, $^6A_{1g} \rightarrow {}^4T_{2g}$ and $^6A_{1g} \rightarrow {}^4t_{1g}$, respectively, indicating octahedral geometry /25/. A weak intensity band exhibited in the region 833-879 nm, which is assigned to the $^5T_{2g} \rightarrow {}^5E_g$ transition, is in fair agreement with hexa-coordinated state for iron(II) complexes /26/.

***Magnetic Moment***

The magnetic moment for the compounds $[Mn(N_4mac^1)Cl_2]$-$[Mn(N_4mac^4)Cl_2]$ observed at 5.12-5.67 B.M. supports an octahedral geometry /27/.

***Mössbauer Spectra***

The Mössbauer spectra of the iron complexes $[Fe(N_4mac^1)Cl_2]$-$[Fe(N_4mac^4)Cl_2]$ have been carried out. The value of isomer shift (0.23-0.38 mm $S^{-1}$) and quardrupole splitting (0.61-0.63 mm $S^{-1}$) at room temperature are characteristic of hexa coordinated iron(II) complexes /28/.

***Mass Spectra***

The mass spectra of the compounds $[Mn(N_4mac^1)Cl_2]$ and $[Fe(N_4mac^2)Cl_2]$ have been recorded. In the mass spectrum of the compound $[Mn(N_4mac^1)Cl_2]$ the molecular ion peak appeared at m/z 592 $[M]^+$. Some other peaks appeared at m/z 594, 516, 491, 460 and 440 corresponding to $M^{+2}$, $[Mn(C_{18}H_{26}O_4H_6)Cl_2]^+$, $[Mn(C_{20}H_{19}O_4N_3)Cl_2]^+$, $[Mn(C_{16}H_{26}O_4N_6)Cl_2]^+$ and $[Mn(C_{12}H_{22}O_4N_6)Cl_2]^+$ species, respectively, which resulted from the loss of the $C_6H_4$, $C_4H_{11}N_3$, $C_8H_4N_2$ and $C_{12}H_8$ fragments from the parent compound, respectively.

In the case of $[Fe(N_4mac^2)Cl_2]$ the molecuar ion peak was observed at m/z 604. The prominent fragments are at 528 for $[Fe(C_{20}H_{14}O_4N_6)Cl_2]^+$, 527 for $[Fe(C_{21}H_{15}O_4N_5)Cl_2]^+$, 472 for $[Fe(C_{16}H_{14}O_2N_6)Cl_2]^+$ and 446 for $[Fe(C_{46}H_8O_4)Cl_2]^+$, due to the loss of $C_6H_4$, $C_5H_3N$, $C_8H_4O_2$ and $C_{10}H_{10}H_6$, respectively.

### *X-Ray Diffraction Spectra*

The lattice dynamics of the products have been ascertained by recording the X-ray diffraction of the compound $[Mn(N_4mac^4)Cl_2]$. The observed 2θ angles, 'd' values and h, k and l values are recorded in Table 3. The data suggested an orthorhombic lattice for this derivative, having unit cell dimensions, a = 32.851, b = 12.990 and c = 19.850.

On the basis of spectral studies it seems that the ligands act as tetradentate chelating agents and the $Cl^-$ anions remained bonded with the metal atom having four coordination sites. Hence a hexa-coordinated environment around the metal atom, assigned in these complexes, is justified.

**Table 3**

X-Ray Powder Diffraction Data of the Compound $[Mn(N_2mac^4)Cl_2]$

| Peak No. | 2θ (Obs.) | 2θ (Calcd.) | d-spacing | h | k | l |
|---|---|---|---|---|---|---|
| 1 | 14.40 | 13.55 | 7.729 | 4 | 0 | 0 |
| 2 | 16.20 | 15.66 | 6.875 | 2 | 1 | 2 |
| 3 | 17.10 | 16.84 | 6.516 | 0 | 0 | 3 |
| 4 | 21.20 | 20.37 | 5.266 | 5 | 0 | 2 |
| 5 | 22.10 | 21.51 | 5.054 | 3 | 1 | 3 |
| 6 | 23.60 | 23.31 | 4.737 | 6 | 0 | 2 |
| 7 | 24.80 | 23.98 | 4.511 | 5 | 0 | 3 |
| 8 | 25.70 | 24.87 | 4.356 | 5 | 2 | 1 |
| 9 | 27.20 | 26.37 | 4.120 | 4 | 0 | 4 |
| 10 | 30.10 | 29.21 | 3.731 | 8 | 1 | 1 |
| 11 | 31.80 | 31.01 | 3.536 | 0 | 3 | 3 |
| 12 | 32.70 | 32.00 | 3.441 | 9 | 1 | 0 |
| 13 | 34.50 | 33.64 | 3.267 | 6 | 3 | 1 |

Refined values : a = 32.851, b = 12.990, c = 19.850

α = β = λ = 90°, max. dev of 2θ = 0.9

### Biochemical studies

### *Antifungal Activities*

The fungicidal action of these complexes has been studied against *Macrophomina phaseolina* and *Aspergillus niger* by spore germination method /29/ and compared with a commercial fungicide bavistin (Table 4). The metal salts have negligible activity, but on complexation are found to be active.

### *Antibacterial Activity*

The title compounds were screened for their antimicrobial activity against gram negative as well as gram positive microorganisms such as *E. coli, S. aureus, S. typhi, B. sublitis, A. aerogenes* and *B. megatherium.* The solvent used was dimethylformamide. Sensitivity plates were seeded with a bacterial inoculum of $1\times10^6$ ciu/mL and each well (diameter 10 mm) was loaded with 0.1 mL of test compound solution of variable concentration in DMF. The zones of inhibition were recorded after incubation for 24 h using *Vernier callibers* (Table 5).

**Table 4**

Antifungal Screening Data of Macrocyclic Complexes

(Average % Inhibition of Spore Germination after 96 hrs)

| Compound | *Macrophomina phasealina* | | | *Aspergillus niger* | | |
|---|---|---|---|---|---|---|
| | 50 ppm | 100 ppm | 200 ppm | 50 ppm | 100 ppm | 200 ppm |
| $[Mn(N_4mac^1)Cl_2]$ | 32 | 46 | 53 | 25 | 52 | 65 |
| $[Mn(N_4mac^2)Cl_2]$ | 34 | 51 | 62 | 32 | 49 | 55 |
| $[Mn(N_4mac^3)Cl_2]$ | 51 | 66 | 69 | 49 | 62 | 69 |
| $[Mn(N_4mac^4)Cl_2]$ | 49 | 60 | 68 | 36 | 54 | 60 |
| $[Fe(N_4mac^2)Cl_2]$ | 27 | 36 | 59 | - | 34 | - |
| $[Fe(N_4mac^3)Cl_2]$ | 36 | 51 | 73 | - | 44 | 65 |
| $[Fe(N_4mac^4)Cl_2]$ | 29 | 42 | 69 | 38 | 57 | 62 |
| Standard (Bavistin) | 82 | 100 | 100 | 86 | 100 | 100 |

Out of the title compounds $[Mn(N_4mac^4)Cl_2]$ and $[Fe(N_4mac^4)Cl_2]$ were found highly active (15 mm zone of inhibition with 50/ug/mL) and moderately active (15 mm zone with 100/ug/mL), respectively. Compounds $[Mn(N_4mac^2)Cl_2]$ and $[Fe(N_4mac^2)Cl_2]$ were moderately active against all the microorganisms except *S. typhi* and *A. aerogenes.*

The comparative activity data of these compounds may be interpreted in term of phenyl group content in the compounds, i.e. the molecule having a four phenyl ring has a higher effect as compared to that which has a two phenyl ring. Further, the biocidal function of these compounds can also be described in terms of chelation theory /22/. Secondly, the activity increases as the concentration increases.

**Table 5**

Antimicrobial Activity of the Macrocyclic Complexes with Different Concentrations (Diameter of Inhibition in mm)

| Compound | *E. coli* | *S. aureus* | *S. typhi* | *B. subtilis* | *A. aerogenes* | *B. megatherium* |
|---|---|---|---|---|---|---|
| $[Mn(N_4mac^1)Cl_2]$ | + | - | + | + | + | - |
| $[Mn(N_4mac^2)Cl_2]$ | + | ++ | + | ++ | ++ | ++ |
| $[Mn(N_4mac^4)Cl_2]$ | +++ | +++ | ++ | ++ | +++ | +++ |
| $[Fe(N_4mac^1)Cl_2]$ | + | - | ++ | + | + | + |
| $[Fe(N_4mac^2)Cl_2]$ | ++ | ++ | ++ | ++ | + | ++ |
| $[Fe(N_4mac^4)Cl_2]$ | +++ | ++ | ++ | +++ | +++ | ++ |

(-) = inactive (less than 12 mm), (+) = weakly active (12-16 mm),

(++) = moderately active (17-20 mm) and (+++) = highly active (21-30 mm)

***Antiinflammatory Activities***

A freshly prepared 1% suspension of carragenine in 0.9% saline was infected under plantar aponeurosis of the right paw of the mice by the reported method /29/. One group of six mice was kept as control and animals of other groups of six each were treated with the test compound in a dose of 50 mg/kg. One group received the standard phenyl butazone. The volume of foot was measured by the micropipette method /30/ and percentage reduction of oedema was calculated at 1h, 8h and 24h.

The anti-inflammatory effect of these compounds was measured against simultaneously run controls using the method of Winter *et al.* /29/. The compounds were found to have significant anti-inflammatory activity at 50 mg/kg dose (Table 6).

**Table 6**

Antiinflammatory Effect of Macrocyclic Complexes on Carragenin Induced Paws Oedema in Albino Mice

| Compound | Percentage reduction of oedema ± SE | | |
|---|---|---|---|
| | 1 h | 8 h | 24 h |
| $[Mn(N_4mac^1)Cl_2]$ | 36.24 ± 0.68 | 67.84 ± 0.81 a | 73.64 ± 1.35 |
| $[Mn(N_4mac^3)Cl_2]$ | 25.77 ± 0.48 | 77.02 ± 0.40 c | 88.36 ± 0.66 b |
| $[Fe(N_4mac^3)Cl_2]$ | 26.13 ± 1.28 | 76.77 ± 0.33 | 85.02 ± 0.61 b |
| $[Fe(N_4mac^4)Cl_2]$ | 23.45 ± 1.88 | 72.16 ± 0.95 c | 83.86 ± 0.54 b |
| Phenyl butazal (Standard) | 29.06 ± 1.96 | 64.66 ± 1.88 b | 80.12 ± 1.70 c |

$p > 0.05$, a : $p > 0.01$, b : $p > 0.005$, c : $p > 0.001$, dose 50 mg/kg

There was no significant reduction in the oedema in all the groups administered with the test drug after 1h ($P < 0.05$). After 8 hours there was a significant reduction in the oedema in most of the groups administered with the test drug. In the case of $[Fe(N_4mac^3)Cl_2]$, $[Mn(N_4mac^3)Cl_2]$ and $[Fe(N_4mac^4)Cl_2]$ the reduction in oedema was highly significant (P > 0.001) as comparable to phenyl butazone (P > 0.005) while in the test compound [Mn($N_4$mac[1])$Cl_2$] the reduction was also significant (P > 0.001), but less than phenyl butazone (P > 0.005). After 24 hours the reduction in oedema in most of the compounds was not very significant except phenylbutazone. The onset of action was found to be after 1 hour and the activity reached its peak after 8 hours. The results suggested that the compounds [Mn($N_4$mac[3])$Cl_2$], [Fe($N_4$mac[3])$Cl_2$] and [Fe($N_4$mac[4])$Cl_2$] have more antiinflammatory activity than standard phenyl butazone.

### *Antifertility Activity*

The reactivity of synthetic products towards biological systems is an important feature of current research and macroocyclic compounds of transition metals play a significant role in this direction. A large number of manganese compounds have been shown to cause atrophy of the testis, prostate and epididymis in male mice /31/. In view of the potential interest in these biologically active compounds, the antifertility activity of some selected compounds has been studied (Table 7) in male mice.

**Table 7**
Antifertility Activity of the Macrocyclic Complexes

| Compound | Sperm motility (%) | Sperm count in cauda epididymis (m/ml) |
|---|---|---|
| Vehicle alone (Olive oil) | 83.4±4.8 | 25.4±3.0 |
| [Mn($N_4$mac[2])$Cl_2$] | 20.8±2.5[a] | 6.0 ± 15[a] |
| [Mn($N_4$mac[3])$Cl_2$] | 13.8±1.3[a] | 3.0±1.0[a] |

Values are expressed as mean ± S.E.
a = (p < 0.001), b = (p < 0.01), c = (p < 0.05)

The colony-bred adult mice were used and 45 male mice (body weight 40-50 g) were divided randomly into three groups of 15 animals each. The animals were kept in plastic cages measuring 25 cm. × 20 cm and only five animals were housed in a cage. The animals were maintained on mice feed pallets (Hindustan Lever Ltd., India) and water was provided *ad libitum*. Only two compounds were used separately and each compound was administered at a dose level of 10 mg/kg wt/day, orally by garage tube for twenty five days. One group served as control and olive oil was used as the vehicle. After 24 h of the last administration, five animals from each group were autopsied and the reproductive organs were removed by dissection, freed from adherent tissues and weighed up to the nearest milligram. The sperm motility and sperm count in cauda epididymis were measured by using Neubaur's hemocytormeter according to the reported method /32/. It was observed that the motility and count of sperm decreased after the administration of the complexes and the spermatogenia and the accessory sex organs were also affected in treated mice. The prostate gland became swollen and the effects did not become normal even after 30 days of recovery, showing the irreversible nature of the effects. A highly significant decline (P < 0.001) in the motility of sperm was observed in the case of [Mn($N_4$MaC[3])$Cl_2$] complexes. The sperm count was also found to decrease significantly in the treated animals. The antifertility activity data indicate that the complexes affect the motility as well as the count of sperm in male mice. Further studies concerning other tests for these complexes are in progress.

**ACKNOWLEDGEMENT**

One of the authors (AC) is thankful to CSIR New Delhi for financial assistance in the form of SRF vide Grant No. 9/149 (288)/2K2, EMR-I.

**REFERENCES**

1. M.W. Hosseini, J.M. Lehn, S.R. Duff, R. Gu and M.P. Mertes, *J. Org. Chem.*, **52**, 1662 (1987).
2. R.M. Izan, J.S. Brandshaw, S.A. Neilsen, J.D. Lamb, J.J. Christensen and D. Sen, *Chem. Rev.*, **85**, 271 (1985).
3. M. Shaker, K.S. Islam, A.K. Mohamed, M. Shagufta and S.S. Hasan, *Trans. Met. Chem.*, **24**, 577 (1999).
4. M.R. Champress, C.S. Frampton, G. Raid and D.A. Tocher, *J. Chem. Soc. Dalton Trans.*, **1994**, 3031.
5. J.J.R. Fransto da Silva and R.J.P. Williams, *The Biological Chemistry of the Elements*, Clarendon Press Oxford, 1991.
6. K.R. Adam, M. Antolovich, D.S. Baldwin, P.A. Duckworth, A.J. Ceong, L.F. Lindoy, M. McParttin and P.A. Taskar, *J. Chem. Soc., Dalton, Trans.*, **1993**, 1013.
7. J.P.L. Cox, K.J. Jankowski, R. Kataky, D. Pasker, M.A. Eaton, M.R. Belley, A.T. Millican, A. Harrison and C. Walker, *J. Chem. Soc. Chem. Commun.*, **1989**, 797.
8. I. Tabushi, Y. Taniguchi and H. Kato, *Tetrahedron Lett.*, **12**, 1049 (1997).
9. M.P. Suh and S.G. Kang, *Inorg. Chem.*, **27**, 2544 (1988).
10. G. Albertin, E. Bordighon and A.A. Orio, *Inorg. Chem.*, **14**, 1411 (1975).
11. K.D. Karlin and J. Zubieta (Eds.), *Copper Coordination Chemistry, Biological and Inorganic Perspective*, Academic Press, New York, 1983; p. 43.
12. A. Singh and Pramila Singh, *Indian J. Chem.* **39A**, 874 (2000).
13. S.G. Shirodkar, P.S. Mane and T.K. Chondhekar, *Indian J. Chem.*, **40A**, 1114 (2001).
14. M. Deveneux, M. McCann, L. Vanessa, M. Geraghty, V. Mckee and J. Wikaira, *Metal Based Drugs*, **7**, 275 (2000).
15. M. McCann, M. Geraghty, M. Devereux, D. O'Shea, J. Mason and L.O'Sullivan, *Metal Based Drugs*, **7**, 185 (2000).
16. N. Fahmi, A. Bansal, S.C. Joshi and R.V. Singh, *Asian J. Chem.*, **11**, 1488 (1999).
17. D.L. Arora, K. Lal, S.P. Gupta and S.K. Sahri, *Polyhedron*, **5**, 1499 (1986).
18. M.B.H. Howlader, M.S. Islam and M.R. Karim, *Indian J. Chem.*, **39A**, 407 (2000).
19. R.K. Agarwal and S.K. Gupta, *Rev. Roum. Chem.*, **32**, 447 (1987).

20. N.B. Cotlhup, L.H. Daly and S.E. Weberly, *Introduction to IR and Raman Spectroscopy*, Academic Press, Inc. New York, 1964.
21. W.U. Malik, R. Bembi and R.D. Singh, *Polyhedron,* **2**, 369 (1983).
22. K. Sharma, S.C. Joshi and R.V. Singh, *Metal Based Drugs,* 7, 237 (2000).
23. M. Shakir, S.P. Varkey and O.S.M. Nasman, *Indian J. Chem.,* **35A**, 671 (1996).
24. A.K. Mohamed and O.S.M. Nasman, *Polyhedron,* **15**, 3487 (1996).
25. M.N. Patel and V.J. Patel, *Synth. React. Inorg. Met-Org. Chem.,* **19** (2), 137 (1989).
26. A.T. Baker, P. Singh and V. Vigncvich, *Aust. J. Chem.,* **44**, 1041 (1971).
27. R.S. Lal, A. Kumar and J. Chakravosty, *Indian J. Chem.,* **40A**, 4222 (2001).
28. H.A. Goodwin, *Coord. Chem. Rev.,* **18**, 314 (1976).
29. G. Seth and N.K. Mourya, *Asian J. Chem.,* **14**, 283 (2002).
30. A. Winter, A. Charles Edwin and W.N. Georg, *PNC Soc. Exptl. Biol. Med.,* **3**, 544 (1992).
31. R.R. Levier and M.E. Jarkowiak, *Biol. Reprod.* 7 (2), 260 (1972).
32. M.R.N. Prasad, N.J. Chinoy and K.M. Kadama, *Fertil. Steril,* **23**, 186 (1972).

